# Regulation of retinal pigment epithelial cell phenotype by Annexin A8

**DOI:** 10.1038/s41598-017-03493-3

**Published:** 2017-07-05

**Authors:** Katharina Lueck, Amanda-Jayne F. Carr, Dimitrios Stampoulis, Volker Gerke, Ursula Rescher, John Greenwood, Stephen E. Moss

**Affiliations:** 1 Department of Cell Biology, UCL Institute of Ophthalmology, 11-43 Bath Street, EC1V 9EL, London, United Kingdom; 2 Department of Ocular Cell Biology and Therapeutics, UCL Institute of Ophthalmology, 11-43 Bath Street, EC1V 9EL London, United Kingdom; 3 Institute of Medical Biochemistry, Centre for Molecular Biology of Inflammation, Muenster, Germany

## Abstract

The retinoic acid derivative fenretinide (FR) is capable of transdifferentiating cultured retinal pigment epithelial (RPE) cells towards a neuronal-like phenotype, but the underlying mechanisms are not understood. To identify genes involved in this process we performed a microarray analysis of RPE cells pre- and post-FR treatment, and observed a marked down-regulation of AnnexinA8 (AnxA8) in transdifferentiated cells. To determine whether AnxA8 plays a role in maintaining RPE cell phenotype we directly manipulated AnxA8 expression in cultured and primary RPE cells using siRNA-mediated gene suppression, and over-expression of AnxA8-GFP in conjunction with exposure to FR. Treatment of RPE cells with AnxA8 siRNA recapitulated exposure to FR, with cell cycle arrest, neuronal transdifferentiation, and concomitant up-regulation of the neuronal markers calretinin and calbindin, as assessed by real-time PCR and immunofluorescence. In contrast, AnxA8 transient over-expression in ARPE-19 cells prevented FR-induced differentiation. Ectopic expression of AnxA8 in AnxA8-depleted cells led to decreased neuronal marker staining, and normal cell growth as judged by phosphohistone H3 staining, cell counting and cleaved caspase-3 levels. These data show that down-regulation of AnxA8 is both necessary and sufficient for neuronal transdifferentiation of RPE cells and reveal an essential role for AnxA8 as a key regulator of RPE phenotype.

## Introduction

Retinal pigment epithelial (RPE) cells and the retina are developmentally derived from the same tissue; the optic vesicle neuroectoderm, and throughout life RPE cells perform a variety of functions to support and protect the retina. These include phagocytosis of photoreceptor outer segments^1^, adsorption of free radicals by pigment granules^2^ and maintenance of ocular immune privilege by forming the outer blood-retina barrier^3^.

Another striking feature of RPE cells, in some species, is their capacity to transdifferentiate into precursor cells and regenerate neuronal tissue. Accordingly, in urodele amphibians such as newts, complete retinal regeneration occurs via RPE transdifferentiation following ocular neuronal injury regardless of life stage^4, 5^. In mammals, however, the ability of RPE cells to transdifferentiate *in vivo* is lost during early embryonic development. Therefore, neuronal cell injury, of the type that occurs in neurodegenerative diseases such as retinitis pigmentosa or age-related macular degeneration, usually results in irreversible vision loss^6, 7^. However, there is evidence that despite being largely post-mitotic, some mature RPE cells continue to divide^8, 9^ mostly in the peripheral retina^10^, as well as during pathological complications following retinal detachment that lead to proliferative vitreoretinopathy^11^. In contrast, when cultured *ex vivo*, RPE cells can be highly proliferative, though this is usually accompanied by substantial de-differentiation manifested as loss of pigment granules, cell polarity and expression of key RPE cell genes such as RPE65 and the Mer tyrosine kinase. Various studies have shown that under suitable conditions, a more authentic RPE cell phenotype can be restored, demonstrating the phenotypic plasticity of these cells in culture^12^. Conversely, RPE transdifferentiation can be induced *in vitro* by basic fibroblast growth factor (bFGF) or retinoic acid (RA)^13–15^, factors known to play a key role in RPE reprogramming during development and retinal regeneration in urodeles^16^.

In this study, the RA derivative Fenretinide (FR) was used to induce transdifferentiation of RPE cells towards a neuronal-like phenotype as described previously^15, 17^. FR exerts its properties in a similar manner to RA; upon binding to nuclear RA receptors (RARs), RARs dimerise with retinoid-X-receptors and activate the RA response element (RARE), leading to transcription of target genes^18–20^. Here we performed a microarray analysis to identify genes involved in the FR-induced transdifferentiation of RPE cells, and observed that AnxA8 was strongly down-regulated upon 7 days exposure to FR. We had a particular interest in AnxA8 and its role in FR-mediated changes, since it was previously associated with osteoclast differentiation^21^. AnxA8 is one of 12 human annexins, most of which share the ability to bind calcium-dependently to negatively-charged phospholipid membranes. Annexins are implicated in cell growth and proliferation^22, 23^, vesicle trafficking^24^, and membrane and cytoskeletal organization^25^. AnxA8 was first identified as vascular anticoagulant-β in the human placenta, where it was described to inhibit blood coagulation and phospholipase A_2_^26^. AnxA8 has been linked with endosome formation in Hela cells^27^, and it plays a role in leukocyte recruitment through exposing cell surface markers on endothelial cells such as CD63 and P-selectin^28^. We show here that suppression of AnxA8 phenocopies the effects of FR, and is both necessary and sufficient to induce neuronal transdifferentiation of RPE cells. These observations identify a novel role for AnxA8 as a key regulator of RPE phenotype.

## Results

### FR and AnxA8 siRNA suppress AnxA8

**Figure 1:**
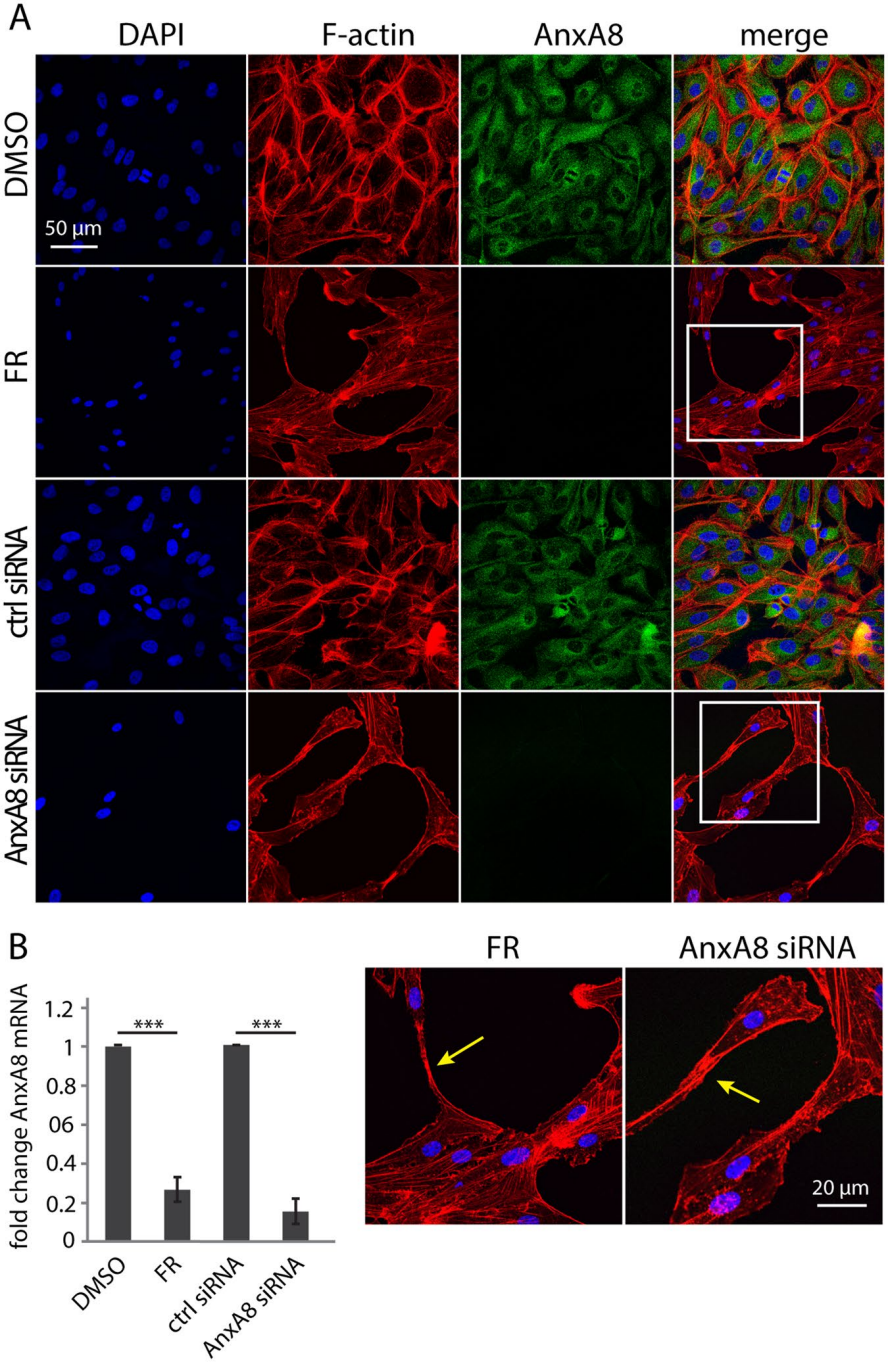
Suppression of AnxA8 phenocopies FR in RPE cells. (**A**) Immunofluorescence staining for AnxA8 (green) is decreased in response to both FR (7 days) and AnxA8 siRNA in ARPE-19 cells whereas vehicle (DMSO) and control siRNA had no effect. Nuclei were stained with DAPI (blue), and rhodamine phalloidin (red) revealed cell extensions in FR- or AnxA8 siRNA-treated cells (yellow arrows in insets). Experiments were performed three times and representative images are shown, scale bar = 50 µm. (**B**) Expression levels of AnxA8 were analysed with real-time PCR. Both FR and AnxA8 siRNA elicited significant reductions in the level of AnxA8 mRNA. Shown are mean and standard error of five independent experiments. Student’s t-test was used to calculate statistical significance (***=P < 0.001).

**Figure 2:**
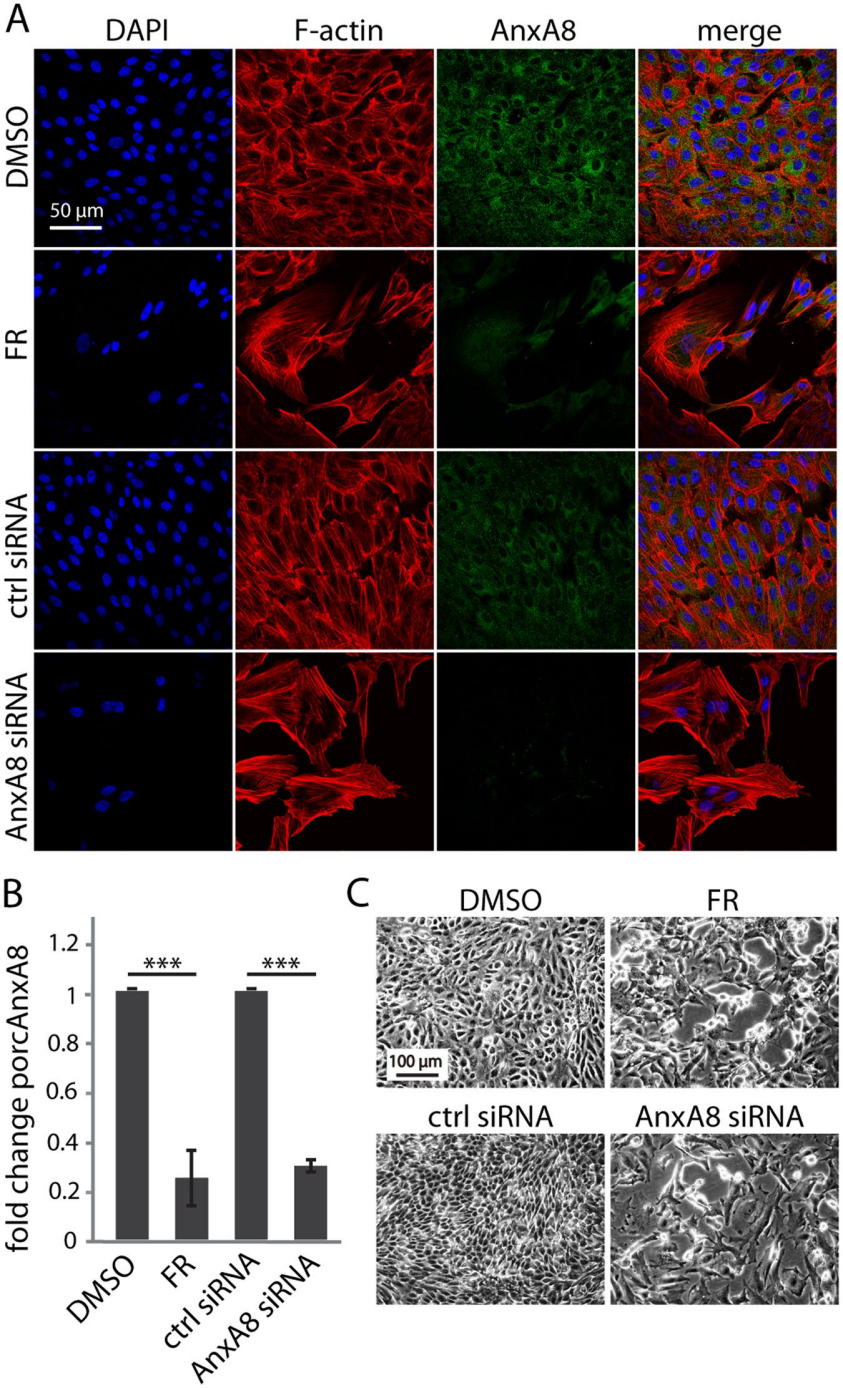
Primary porcine RPE respond to FR and AnxA8 siRNA in the same way as ARPE-19 cells. (**A**) FR and AnxA8 siRNA treatment for 7 days results in fewer cells (DAPI, blue), formation of cytoplasmic extensions and F-actin rich domains at the cell borders (rhodamine phalloidin, red) with concomitant down-regulation of AnxA8 (green). Scale bar = 50 μm. Shown are representative immunofluorescence images from 3 independent experiments. (**B**) Cell lysates were prepared under each of the conditions shown in (**A**) and AnxA8 siRNA levels were quantified by real-time PCR. Similar to ARPE-19 cells, porcine AnxA8 mRNA transcripts were down-regulated in the presence of FR and AnxA8 siRNA. Data show mean and standard error from 5 independent experiments. Student’s t-test was used to calculate statistical significance (***=**P** < 0.001). (**C**) Representative phase images show that FR and AnxA8 siRNA treatment both lead to extension formation and suppression of cell growth as observed in ARPE-19 cells. Scale bar = 100 μm.

We undertook a microarray analysis of FR-treated ARPE-19 cells in order to identify genes that might mediate the effects of FR. As expected, and consistent with published observations^15, 17^, we observed an increase in the expression of the neuronal marker calretinin in response to FR, and strong down-regulation of AnxA8, a gene which has been linked with cell differentiation processes^21^ (Table 1). To validate the microarray data, we performed immunofluorescence analysis of AnxA8 in FR- and dimethyl sulfoxide (DMSO) control-treated cells, which showed that FR treatment led to almost complete disappearance of AnxA8 staining in both ARPE-19 cells (Fig. 1A) and primary porcine RPE (pRPE) cells (Fig. 2A). Real-time polymerase chain reaction (PCR) analysis revealed a ~70% down-regulation of AnxA8 expression in both FR-treated ARPE-19 (Fig. 1B) and pRPE cells (Fig. 2B). To elucidate whether AnxA8 has a causative role in transdifferentiation or is suppressed as a consequence, short interfering ribonucleic acid (siRNA) was used to suppress AnxA8 gene expression in RPE cells. Immunostaining revealed that following siRNA treatment, AnxA8 was barely detectable in both ARPE-19 (Fig. 1A) and pRPE cells (Fig. 2A). Furthermore, DAPI and phalloidin staining showed a marked reduction in cell number and the formation of extensions in the absence of AnxA8 that resembled those observed with FR.

**Table 1:** Microarray data pre- and post-FR treatment showing 40 of the most up- and down-regulated genes and their fold change in response to FR versus control treatment. AnxA8 was one of the most down-regulated genes, while AnxA7 was almost unchanged. The neuronal marker calretinin was up-regulated in FR-treated ARPE-19 cells.

Probe ID	Gene symbol	Gene name	Fold change	P value
206658_at	UPK3B	uroplakin 3B	−6.94642	1.38E-07
206758_at	EDN2	endothelin 2	−5.78156	2.48E-08
211029_x_at	FGF18	fibroblast growth factor 18	−5.67398	7.78E-10
219054_at	C5orf23	chromosome 5 open reading frame 23	−5.44576	5.92E-08
211959_at	IGFBP5	Insulin-like growth factor binding protein 5	−5.27773	4.60E-06
**203074_at**	**ANXA8**	**annexin A8**	**−4.63574**	**5.14E-10**
219790_s_at	NPR3	natriuretic peptide receptor	−4.3291	1.96E-07
219937_at	TRHDE	thyrotropin-releasing hormone degrading enzyme	−3.9654	1.49E-07
223063_at	C1orf198	chromosome 1 open reading frame 198	−3.75853	2.50E-08
201564_s_at	FSCN1	fascin homolog 1	−3.67614	2.37E-07
209292_at	ID4	Inhibitor of DNA binding 4	−3.6008	2.31E-09
204451_at	FZD1	frizzled homolog 1 (Drosophila)	−3.28944	4.37E-08
228885_at	RPL24	ribosomal protein L24	−3.24399	1.39E-07
218931_at	RAB17	RAB17, member RAS oncogene family	−3.20924	2.18E-09
20364_s_at	COBLL1	COBL-like 1	−3.18439	1.64E-07
207787_at	KRT	keratin 33B	−3.14621	2.54E-07
217841_s_at	PPME1	protein phosphatase ethyl esterase 1	−2.7241	1.16E-07
240890_at	CASP4	caspase 4	−2.63728	3.68E-09
212158_at	SDC2	syndecan 2	−2.55582	9.17E-08
209082_s_at	Col18A1	collagen type XVII	−2.01389	1.19E-07
**201366_at**	**ANXA7**	**annexin A7**	**1.38439**	**5.73E-06**
**205428_s_at**	**CALB2**	**calbindin 2 (calretinin)**	**2.87484**	**1.24E-05**
209278_s_at	TFPI2	tissue factor pathway inhibitor 2	2.93228	5.23E-08
201425_at	ALDH2	aldehyde dehydrogenase 2 family	2.94253	1.27E-07
214453_s_at	IFI44	interferon-induced protein 44	3.172	7.16E-07
205110_at	FGF13	fibroblast growth factor 13	3.25386	1.78E-08
205047_at	ASNS	asparagine synthetase	3.30353	4.79E-08
223062_s_at	PSAT1	phosphoserine aminotransferase 1	3.31132	7.38E-07
214430_at	GLA	galactosidase, alpha	3.39277	2.68E-06
205730_s_at	ABLIM3	actin binding LIM protein family, member 3	3.45524	6.46E-07
205479_s_at	PLAU	plasminogen activator, urokinase	3.51411	1.38E-06
238133_at	NTNG1	netrin G1	3.52826	1.64E-06
213112_s_at	SQSTM1	sequestosome 1	3.87001	1.03E-06
208025_s_at	HMGA2	high mobility group AT-hook 2	4.14352	5.32E-07
212298_at	NRP1	neuropilin 1	4.62486	4.84E-06
203505_at	ABCA1	ATP-binding cassette	5.71048	7.09E-08
202859_x_at	IL8	interleukin 8	6.38496	1.64E-07
211506_s_at	IL18	interleukin 18	6.42257	1.07E-08
206172_at	IL13RA2	interleukin 13 receptor, alpha 2	6.44241	2.16E-07
219926_at	POPCD3	Popeye domain containing 3	6.82434	8.44E-08

### FR and AnxA8 siRNA induce a neuronal-like phenotype

**Figure 3:**
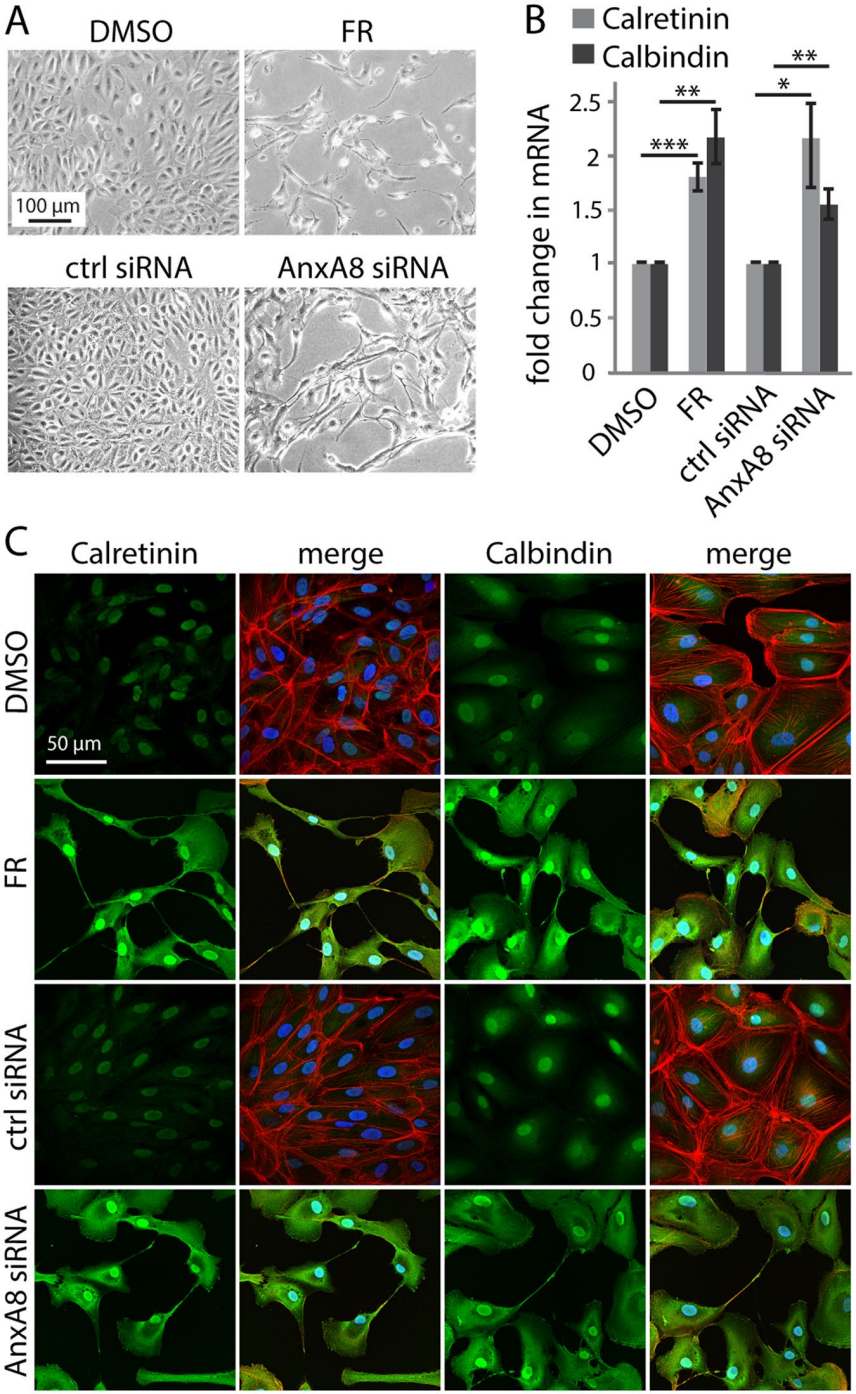
Loss of AnxA8 leads to up-regulation of neuronal markers in RPE cells. (**A**) Phase images of FR- or AnxA8 siRNA-treated ARPE-19 cells show reduced cell growth and formation of neuronal-like extensions compared to respective controls. Experiments were performed three times and representative images are shown, scale bar = 100 μm. (**B**) mRNA was extracted from ARPE-19 cells following treatment with FR or AnxA8 siRNA and subjected to real-time PCR analysis for calretinin and calbindin. The results show that both genes were significantly up-regulated by FR and AnxA8 siRNA (** = **P** < 0.01, *** = **P** < 0.001, n = 4). (**C**) ARPE-19 cells were treated with FR and AnxA8 siRNA, then fixed, immunostained and visualized by confocal microscopy. Calretinin and calbindin (both green) were stained along with nuclei (DAPI, blue) and F-actin (rhodamine phalloidin, red). Calretinin and calbindin staining in cells treated with FR and AnxA8 siRNA was markedly enhanced and more evident in the cytosol, scale bar = 50 μm.

To further examine the role of AnxA8 in determining RPE phenotype, we used phase images of FR- or AnxA8 siRNA-treated RPE cells to visualise cellular morphology. As reported previously^15, 17^, FR induced an arrest in cell growth and formation of neuron-shaped extensions in ARPE-19 cells, whereas DMSO control-treated cells showed a more normal epithelial phenotype and higher cell density. Similar morphological changes were observed after siRNA-mediated AnxA8 depletion (Fig. 3A), and pRPE cells displayed comparable features in response to FR and AnxA8 siRNA treatment regarding cell morphology changes (Fig. 2C). Furthermore, the neuronal markers calretinin and calbindin were significantly upregulated to a similar extent in both FR- and AnxA8 siRNA-treated ARPE-19 cells as demonstrated by real-time PCR (Fig. 3B). Immunofluorescence imaging revealed a marked increase in staining intensity for calretinin and calbindin upon exposure to FR and AnxA8 siRNA, as well as a change in subcellular localisation from exclusively nuclear to both nuclear and cytosolic. The staining also emphasises the formation of the characteristic neuronal-like extensions between the cells, and changes to the F-actin cytoskeleton (Fig. 3C). To determine whether the acquisition of neuronal characteristics was accompanied by loss of hallmark RPE features, we examined the expression of a number of RPE cell markers (retinaldehyde binding protein 1, keratin 8, bestrophin 1, tyrosinase, tyrosinase related protein 1, premelanosome protein, monocarboxylate transporter 3, mer tyrosine kinase, paired box 6, orthodenticle homeobox 2, growth arrest specific 1, melanogenesis associated transcription factor) in FR- or AnxA8 siRNA treated ARPE-19 cells. However, without exception these were either expressed at very low levels or undetectable (results not shown), consistent with our previous study^15^. Taken together with the data in Fig. 1, these observations show that down-regulation of AnxA8 is sufficient to induce neuronal characteristics in RPE cells.

### AnxA8 over-expression inhibits FR-induced changes in morphology

**Figure 4:**
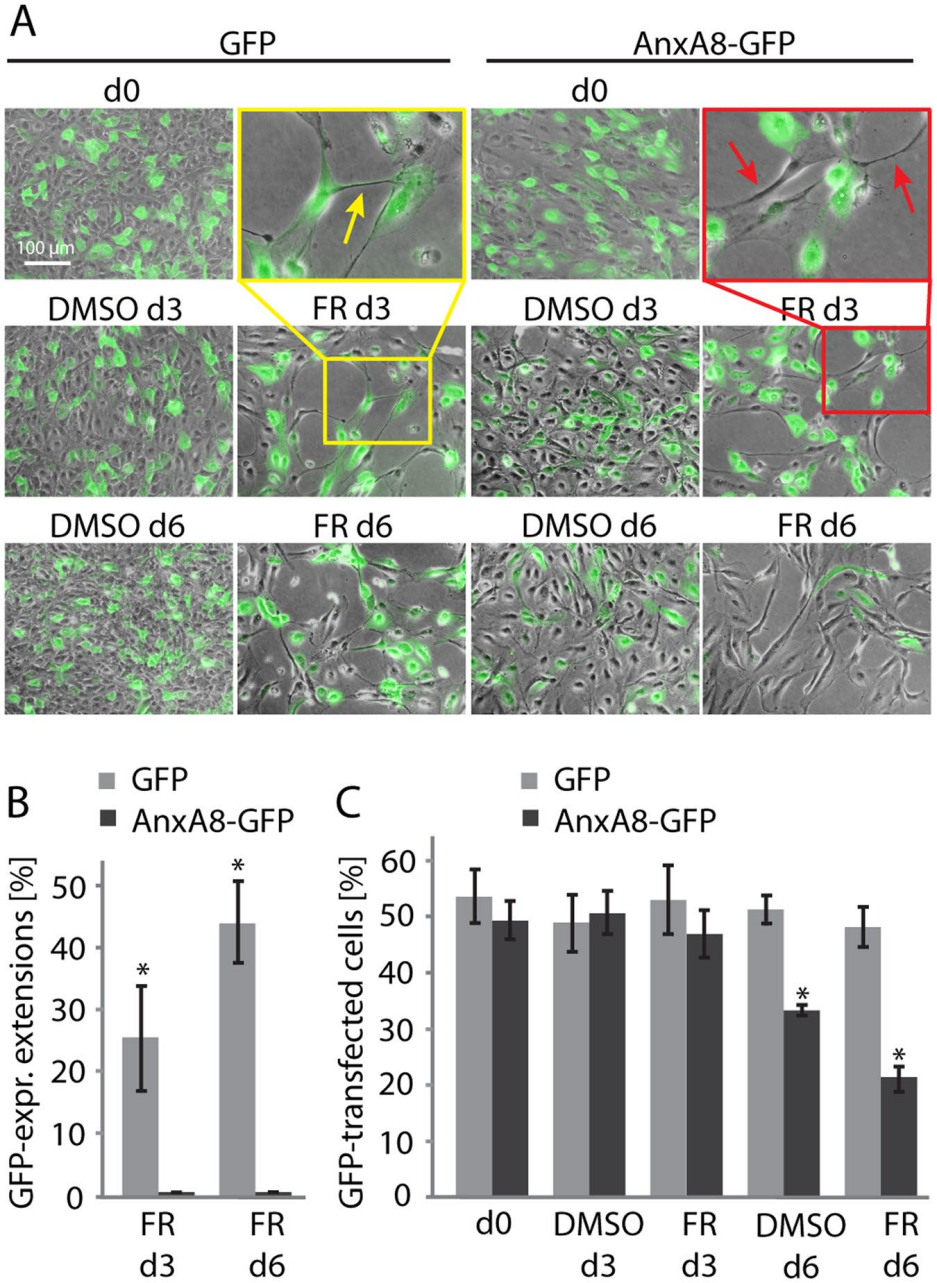
Ectopic expression of AnxA8 prevents FR-induced neuronal transdifferentiation. (**A**) Merged phase and fluorescence images of AnxA8-GFP- and GFP-transfected ARPE-19 cells, treated with either FR or the DMSO control for 0, 3 and 6 days. FR-induced transdifferentiation was observed in GFP-transfected (yellow arrow in inset) or non-transfected cells only (red arrows in inset). (**B**) Quantification of ARPE-19 cell extensions revealed 25% and 43% of GFP transfected extensions on day 3 and day 6 respectively of FR treatment, while AnxA8-GFP was never found in FR-induced cell extensions. Six representative images from four independent experiments were counted. (**C**) Quantification of the number of transfected cells at different stages of the experiment revealed that the initial transfection rate of the starting transfection rate of ~50% was maintained in GFP transfected cells after 6 days, whereas the proportion of AnxA8-GFP-transfected cells diminished to ~32% in DMSO and ~20% in FR. Data are mean and standard error of four independent experiments. For each experiment cells from 6 representative images were counted. Student’s t-test was used to calculate statistical significance (* = *P* < 0.05).

We next wanted to find out whether suppression of AnxA8 was necessary for the FR-induced transdifferentiation of RPE cells. For these experiments we performed transient ectopic expression of AnxA8-GFP or GFP (as control) in RPE cells immediately prior to treatment with either FR or DMSO (Fig. 4A). By day 3 of FR or DMSO treatment, FR induced neuronal-like transdifferentiation of control GFP-transfected cells as was seen in neighbouring non-transfected cells (yellow arrow). The respective DMSO control had no effect on the cell morphology of either AnxA8-GFP- or GFP-transfected cells. However, when exposed to FR for 3 days, cells transfected with AnxA8-GFP remained in an undifferentiated state with no extension formation, while adjacent, non-transfected cells exhibited the characteristic neuronal-like morphological changes (red arrows). The percentage of GFP-expressing extensions was 25% on day 3 of FR treatment, and 43% on day 6, consistent with the on-going acquisition of neuronal characteristics in the growth-arrested cells. In contrast, AnxA8-GFP-transfected cells never formed extensions when treated with FR (Fig. 4B). By day 6, the relative number of cells expressing control GFP was unchanged from day 0 whether cells were treated with DMSO or FR (Fig. 4C). However, by day 6 the percentage of AnxA8-GFP positive cells diminished in control and FR-treated cells to 34% and 22% respectively, suggesting that together with the loss of expression one would anticipate in a transient transfection study, sustained over-expression of AnxA8-GFP may not be well tolerated by RPE cells, particularly in the presence of FR.

### AnxA8 over-expression inhibits FR-induced changes in gene expression

**Figure 5:**
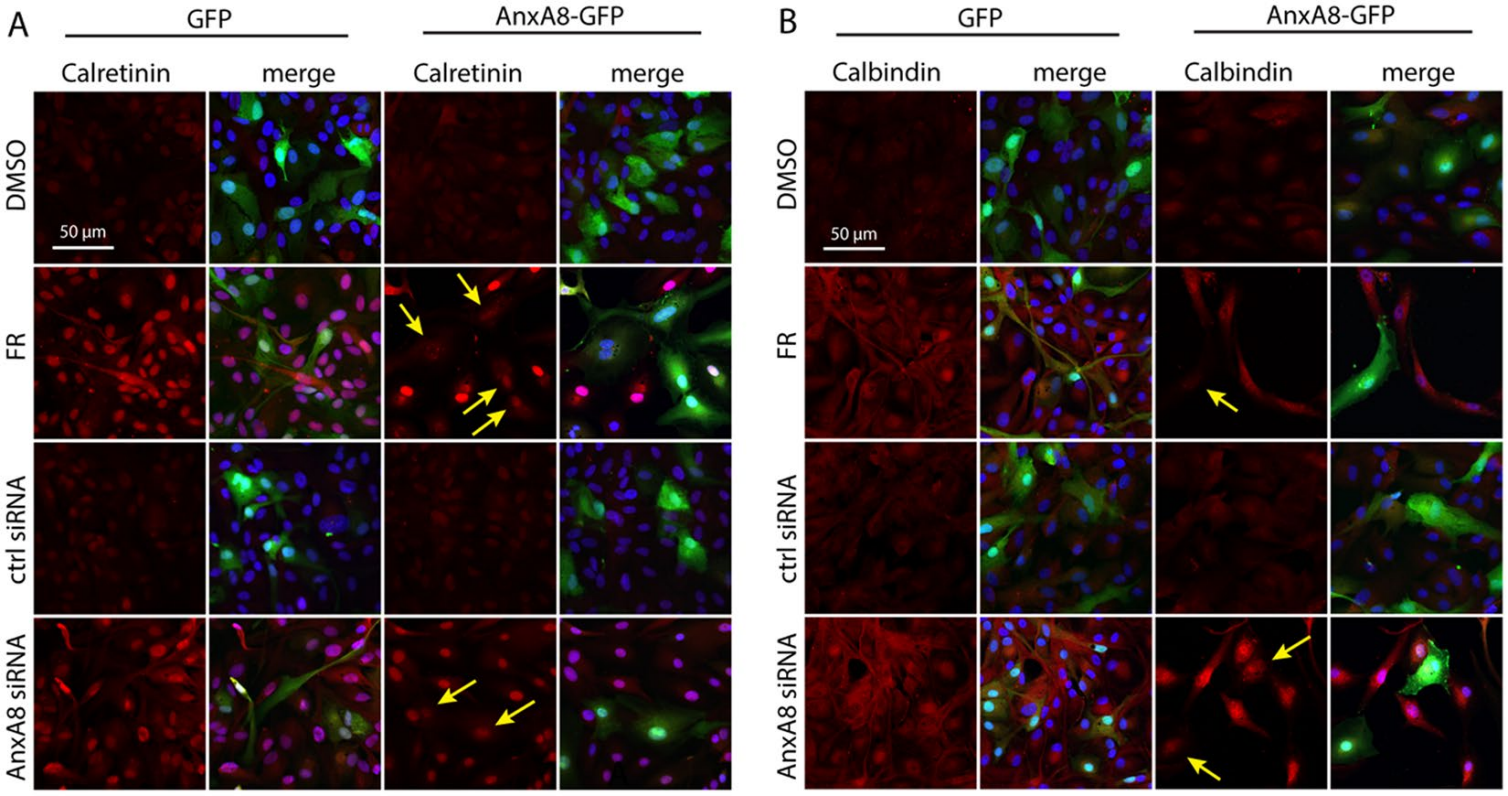
Suppression of AnxA8 is required for induction of calretinin and calbindin. (**A**) Cells were transfected with either GFP or AnxA8-GFP and then treated with FR or AnxA8 siRNA (with appropriate controls) for 3 days. Images show elevated staining for calretinin (red) upon exposure to FR and AnxA8 siRNA, with the merged images showing GFP (green) and nuclei (DAPI, blue). In cells transfected with AnxA8-GFP we observed many instances where there was no apparent increase in expression of calretinin (yellow arrows) following FR or AnxA8 siRNA when compared to DMSO control and control siRNA treatments. Presented are representative images from three independent experiments, scale bar = 50 μm. Similar analysis of calbindin (**B**) in the same set of experimental conditions revealed patterns of expression almost identical to those described above for calretinin.

We also wanted to determine whether forced expression of AnxA8-GFP would prevent the increase in expression of calbindin and calretinin observed upon treatment of ARPE-19 cells with FR or AnxA8 siRNA. Cells were transfected with GFP or AnxA8-GFP, and then cultured in FR or treated with AnxA8 siRNA for 3 days (with appropriate controls) prior to analysis by confocal microscopy. Treatment with both FR and AnxA8 siRNA led to a reduction in cell proliferation in GFP-transfected cells, with concomitant formation of neuronal extensions and increased staining intensity for calbindin and calretinin (Fig. 5A,B). In contrast, in cultures transfected with AnxA8-GFP and then treated with FR or AnxA8 siRNA, most of the transfected cells exhibited markedly reduced staining intensity for calretinin (yellow arrows in Fig. 5A) and calbindin (yellow arrows in Fig. 5B) than their non-transfected neighbours. In these experiments the ectopic expression of AnxA8-GFP was sufficiently high as to be unaffected by the siRNA.

### AnxA8 siRNA and FR induce cell cycle arrest and apoptosis

**Figure 6:**
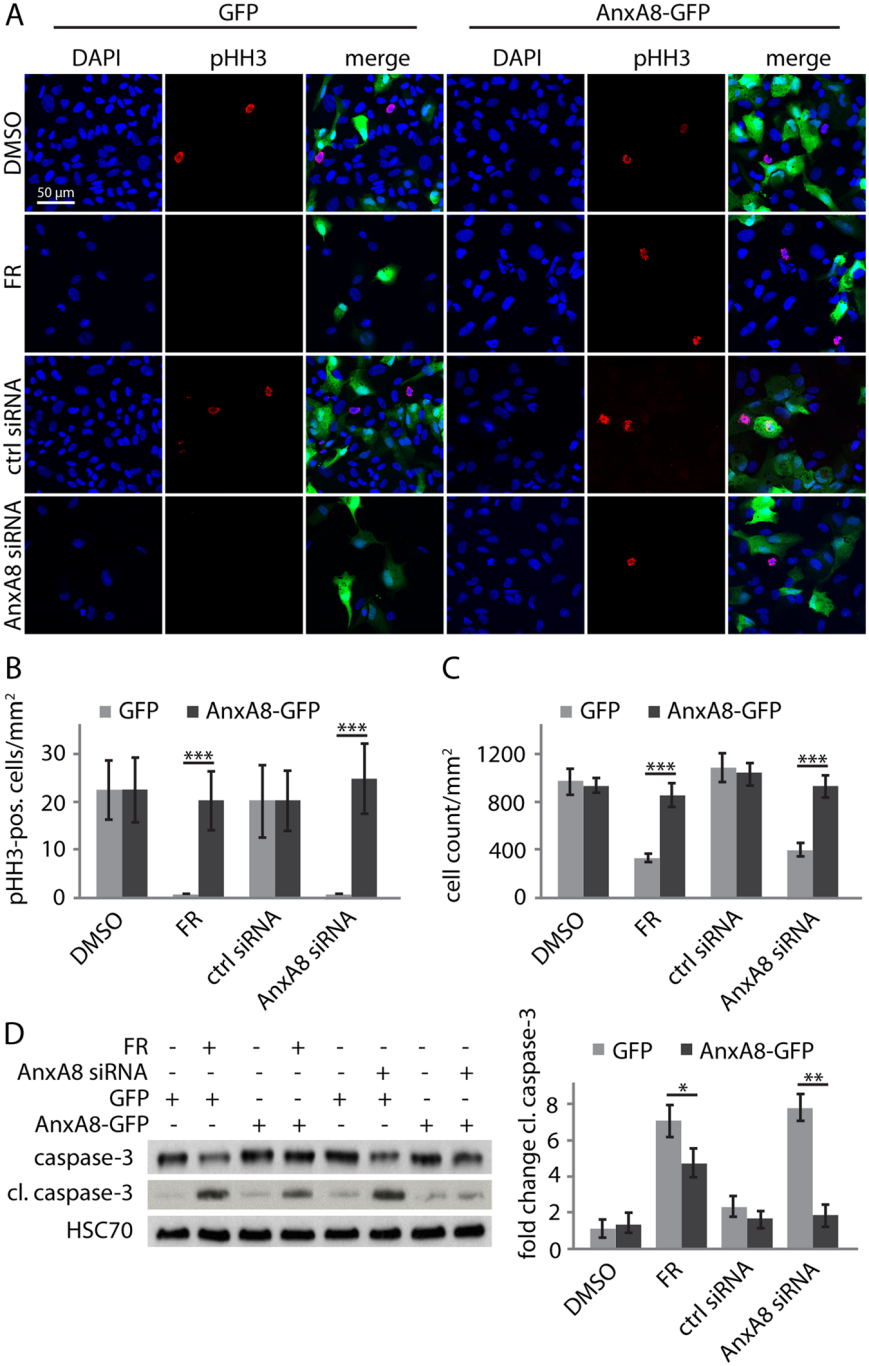
Cell cycle arrest and apoptosis in the presence of FR requires suppression of AnxA8. (**A**) ARPE-19 cells were transfected with GFP or AnxA8-GFP, then treated with FR or AnxA8 siRNA for 3 d before being fixed and immunostained for phosphohistone H3 (pHH3). GFP-transfected ARPE-19 cells showed a reduction in the number of nuclei (DAPI, blue) and in contrast to confluent control samples, almost no cells were positive for pHH3 (red) when treated with FR or AnxA8 siRNA. In AnxA8-GFP-transfected cells, similar numbers of pHH3 positive cells were observed in all conditions. Scale bar = 50 μm. (**B**) Quantitation of pHH3-positive cells under each of the conditions shown in (**A**) employed data from 4 individual experiments, using 5 representative areas for counting. The number of pHH3 positive cells was significantly reduced in GFP-expressing cells in the presence of FR or AnxA8 siRNA and this was completely reversed by expression of AnxA8-GFP. (**C**) Total cell number followed a similar pattern to the proportion of dividing cells, with fewer cells per mm^2^ following FR or AnxA8 siRNA, and reversal of this effect by ectopic expression of AnxA8-GFP. (**D**) While caspase-3 was slightly diminished in control GFP-transfected ARPE-19 cells treated with either FR or AnxA8 siRNA, there was a significant increase in cleaved caspase-3 in these samples as judged by western blotting (cropped images). Mean and standard deviations were obtained from 3 independent experiments. Student’s T-test was used to calculate statistical significance (* = P < 0.05, ** = P < 0.01, *** = P < 0.001).

Finally, we performed experiments to quantify the effects of neuronal transdifferentiation on cell proliferation, since the results of the earlier studies suggested that FR and AnxA8 siRNA both induced cell growth arrest in ARPE-19 and pRPE cells. ARPE-19 cells were transfected with GFP or AnxA8-GFP and then cultured in FR or treated with AnxA8 siRNA for 6 days before being fixed and immunostained for the mitotic marker pHH3 (Fig. 6A). Quantification of the number of pHH3^+^ cells in the GFP-transfected cultures confirmed the near complete absence of mitotic cells in the presence of FR or following AnxA8 siRNA treatment, whereas ectopic expression of AnxA8-GFP restored the number of pHH3^+^ cells to control levels (Fig. 6B). We also counted total cell numbers under each of these experimental conditions to demonstrate that the absence of mitotic cells in the FR- and AnxA8 siRNA-treated cultures was not due to a lack of cells. This analysis revealed that in GFP control transfected cultures the total cell number after FR and AnxA8 siRNA treatment was around one third of that in cultures transfected with AnxA8-GFP (Fig. 6C). We also performed western blotting analysis to determine whether AnxA8-loss induced apoptosis. Control GFP-transfected ARPE-19 cells treated with FR and AnxA8 siRNA showed a significant increase in cleaved caspase-3 (Figs 6D and S1). AnxA8-GFP transfected cells, however, exhibited lower levels of cleaved caspase-3 and rescued caspase-3 levels in FR- or AnxA8 siRNA-treated cells.

## Discussion

RPE cells perform a range of unique functions to support and protect photoreceptor cells and maintain the visual cycle^29^. These highly specialised properties require RPE cells to be terminally differentiated *in vivo*. In most mammalian species it is generally considered that mature RPE cells are post-mitotic, with little evidence for self-renewal or regression to a precursor cell stage^11^. In contrast, RPE cells *in vitro* tend to spontaneously de-differentiate and have been shown to acquire some of the characteristics of other retinal cell types, including ganglion, amacrine, photoreceptor, and glial cells^30, 31^. This apparent plasticity of RPE cells is also supported by evidence that they can be induced to transdifferentiate towards a neuronal-like phenotype^15, 17^. These observations reveal an inherent stemness in RPE cells more commonly seen in newts and other urodeles that have the capacity for retinal regeneration *in vivo*. Interestingly, the constraints on RPE cell division in mammals appear to be lifted under certain pathological conditions such as retinal detachment^32^ or tumour growth^33^.

To gain insight into the molecular mechanisms that regulate RPE phenotype we exploited the established model of FR-induced neuronal transdifferentiation^15, 17^. Microarray analysis of gene expression pre- and post-FR revealed AnxA8 to be among the most strongly down-regulated genes in transdifferentiated RPE cells. AnxA8 is specifically expressed in cells of patients with acute promyelocytic leukaemia (APL)^34–36^, indicating a role in differentiation of haematopoietic cells. Elevated AnxA8 expression levels are associated with terminal osteoclast differentiation^21^, and AnxA8 was recently documented to be present in mouse mammary ductal epithelium^37^. Transcriptomic analysis of human primary RPE also revealed the presence of AnxA8^38^, consistent with its expression in ARPE19 cells observed here.

To address this question, AnxA8 was targeted in RPE cells using siRNA. Interestingly, loss of AnxA8 revealed almost identical phenotypic changes to those induced by FR, including reduced cell proliferation, formation of neuronal-like extensions and concomitant upregulation of neuronal markers. FR has been shown before to increase the expression of neurofilament proteins NF160 and NF200, and induce strong immunostaining of calretinin^15, 17^. A reduction in cell number and formation of extensions have also been reported in endothelial and tumour cells after exposure to FR^39, 40^.

These experiments showed that suppression of AnxA8 is sufficient to cause acquisition of a neuronal phenotype. But we also wanted to know whether suppression of AnxA8 was necessary for the FR-induced phenotypic switch. We therefore transiently over-expressed AnxA8-GFP while exposing the cells to FR. Within the mixed population of transfected and non-transfected cells, those over-expressing AnxA8 were resistant to FR-or AnxA8 siRNA-induced changes. This shows that ectopic expression of AnxA8-GFP can substitute for endogenous AnxA8 and preserve RPE phenotype. However, we also observed a greater time-dependent decrease in the number of AnxA8-GFP-transfected cells in FR-treated versus control cells, suggesting that AnxA8-GFP expression is less well tolerated in the presence of FR and/or the transient expression ceased.

Our findings reveal that AnxA8 is required for maintenance of a normal RPE phenotype, and that loss of AnxA8 may result in RPE reprogramming towards neuronal-like cells. It is interesting to note that AnxA8 has previously been described as a marker of terminal differentiation of osteoclasts^21, 41^. AnxA8 was also shown to modulate proliferation in the mammary gland epithelial cell line Kim-2, in studies in which ectopically expressed AnxA8 triggered senescence-associated changes^37^. Our data indicate that FR regulates cell growth arrest, cell cycle arrest, apoptosis and trans-differentiation of RPE cells with associated up-regulation of the neuronal markers calretinin and calbindin, via down-regulation of AnxA8. Consistent with this assumption are data that showed down-regulation of AnxA8 by retinoic acid to be associated with maturation and differentiation of the APL-derived model cell line NB4^42^. AnxA8 expression was completely suppressed in response to all-trans RA, with reduced proliferation and complete regression of APL, accompanied by an inverse correlation between RAR-α and AnxA8 expression^34, 36^. Changes in RPE phenotype associated with AnxA8 loss might therefore be mediated through the RA signalling pathway, and a decrease in AnxA8 might be necessary for this pathway to be activated.

Although AnxA8 seems to play a major role in maintaining RPE cell phenotype and its suppression leads to neuronal trans-differentiation in culture, its role *in vivo* and its potential interaction with other proteins remains unresolved. In addition, caution is required in extrapolating findings obtained in an *in vitro* culture model to the complex environment of the living retina. In future studies it will be of interest to examine potential interactions between AnxA8 and other developmental factors, such as Wnt signaling genes, sonic hedgehog or the bone morphogenetic proteins, all of which are involved in retinal regeneration^43–45^. In addition to cell-intrinsic factors, choroidal tissue may be important for RPE cell transdifferentiation into neurons, suggesting that interactions between RPE cells and their environment may also stimulate transdifferentiation^46^. This idea is supported by work showing that laminin in the vascular membrane of the choroid can promote RPE transdifferentiation^47^. Essential factors mediating these choroid-RPE interactions in newts as well as chick embryos are FGF-2 and IGF-1^46, 48^, which were detected in RPE cells and in macrophages migrating into the eye cavity in the early stages of retina regeneration^49^. Further, gene transcripts encoding FGF receptors were discovered in RPE cells of chick and mammalian embryos^50, 51^. In summary, we have shown that the well-documented effects of FR on RPE cell phenotype are potentially mediated through down-regulation of AnxA8, and that this is both necessary and sufficient for neuronal transdifferentiation. In the context of tissue regeneration in urodeles it is intriguing to note that among all vertebrates, this is the only order that contains orthologues of the complete mammalian annexin gene family, yet lacks AnxA8.

## Methods

### Isolation and cultivation of porcine RPE

Pig eyes were obtained from Link (Wolverhampton, UK) and kept on ice during transport and preparation. Eyes were disburdened from muscle tissue, cleaned with videne (Ecolab) and phosphate-buffered saline (PBS) 1:5 and incubated in 0.2 mg/ml penicillin/streptomycin for 30 min at 4 °C. Under sterile conditions, the eyes were circularly cut underneath the *Ora serrata*. The neuroretina was detached from the subjacent RPE cells and disconnected from the optical nerve. RPE cells were incubated with 10× trypsin-ethylene diamine tetra acetic acid (EDTA, Gibco) for 20 min at 37 °C and detached by pipetting up and down. Cells were centrifuged at 400× g for 5 min and plated in DMEM and 10% FBS.

### Cell culture

Human ARPE-19 cells (ATCC number CRL-2302)^52^ and primary RPE (pRPE) cells from pigs were cultured in Dulbecco’s modified Eagle’s medium and Ham’s F12 1:1 (DMEM/F12, Gibco) at 37 °C with 5% carbon dioxide. The media was supplemented with 100 U/ml penicillin/streptomycin (Gibco) and 10% foetal bovine serum (FBS, Gibco).

### FR treatment

Cells were seeded into flat-bottom 6-well dishes at 2,200 cells/cm^2^ and allowed to attach overnight. DMEM and 3% charcoal dextran-treated FBS (Thermo Scientific Hyclone) were added for another 24 h to adsorb hormones and growth factors. Cells were then treated with 1 µM FR (Tocris, Bioscience) or DMSO (Sigma) as control in DMEM and 1% FBS every day for 7 days. Subsequently, phase images were obtained (Leica DC200) and cells were processed for real-time PCR and immunofluorescence staining.

### Microarray Analysis

Total RNA was extracted from cells following 7 days treatment with DMSO or Fenretinide, using TriZol reagent (Life Technologies) and purified (RNeasy Mini Kit, Qiagen) according to the manufacturers instructions. cDNA was synthesised using Superscript II and E.Coli DNA polymerase according to the manufacturers instructions (Life Technologies), purified (GeneChip® Sample Cleanup Module, Qiagen) and used as a template for the synthesis of biotin-labelled cRNA (GeneChip IVT Labeling Kit (Affymetrix). The biotin-labelled cRNA was purified and fragmented using the GeneChip® Sample Cleanup Module. cRNAs were hybridised to the Human U133A 2.0 Affymetrix Microarray. Data analysis was performed in R using packages from Bioconductor^53^. Data sets for each array were quality assessed using affyQCReport. GenChip Robust Multiarray averaging (GC-RMA) was used to correct background, normalise data and convert fluorescence into expression levels. Differentially expressed genes were identified using a two sample T-test followed by FDR analysis.

### Suppression of AnxA8

RPE cells were plated in full media and allowed to attach. SiRNA transfection was carried out at 80% cell confluence using a pool of AnxA8 siRNA (Life Technologies) with the following sequences: human AnxA8 siRNA-1: 5′-CAG CCU UUC GGU CUU CUA UTT-3′, human AnxA8 siRNA-2: GCG UGA UGG GAC CCU GAU ATT-3′, human AnxA8 siRNA-3: 5′-GCC CUU AUG UAC CCG CCA UTT-3′^54^. Cells were transfected using Interferin (Polyplus transfection) for 48 h, and each AnxA8 siRNA was added at 150 nM. Allstars siRNA (Qiagen) was used as a control. Cells were washed and incubated with full media for another 48 h. Phase images were taken, and cells were processed for real-time PCR and immunofluorescence analysis.

### Immunofluorescence staining and confocal microscopy

Cells grown on glass coverslips were washed with PBS after FR or AnxA8 siRNA treatment, and were then fixed in 4% paraformaldehyde (PFA) for 10 min at 4 °C. Cells were incubated in 0.1% Triton X100 (Sigma) for another 10 min, and non-specific binding sites were blocked with PBS and 1% BSA for 30 min. Polyclonal rabbit antibodies against human AnxA8 (1:200, AP00292PU-N, Acris), calretinin (1:200, Millipore), calbindin (1:200, Swant, Switzerland) and phosphohistone H3 (pHH3, 1:200, Cell Signalling) were applied overnight at 4 °C. A secondary Alexa fluor 488 donkey anti-rabbit antibody (1:200, Invitrogen) and rhodamine phalloidin (1:200, Invitrogen) were added for 1 h. Nuclei were stained with DAPI (1:1000, Sigma). As a negative control, the primary antibody was omitted. Cells were then mounted in moviol (Calbiochem), and analysed using confocal microscopy (Leica TCS SP2, LSM Zeiss 710). Images were processed in ImageJ (NIH freeware).

### Real-time PCR

Total RNA was isolated from ARPE-19 and pRPE cells using the RNeasy Mini Kit (Qiagen). RNA concentration was measured with a Nanodrop 1000 Spectrophotometer (Thermo Scientific), and the Quantitect RT Kit (Qiagen) was used to reverse transcribe up to 1 µg RNA into cDNA. Real-time amplification was performed on a HT7900 Fast Real-Time PCR System (Applied Biosystems). PCR reactions were performed in triplicates with Power Sybr Green master mix (Life Technologies) containing 1 µl cDNA and 0.3 µM gene-specific primers (Life Technologies, Table 2). Raw data were transferred into the Dart-PCR spreadsheet (version 1.0)^55^, and gene expression was normalised to glyceraldehyde-3-phosphate dehydrogenase (GAPDH). FR and AnxA8 siRNA treatments were normalised to corresponding controls (DMSO/ctrl siRNA). Dissociation curves were generated to assess binding specificity of each primer pair.

**Table 2:** Human and porcine primer pairs used for real-time PCR. Shown are the forward (F) and reverse (R) sequence of the primers, as well as PCR conditions.

Target		Sequence from 5′ to 3′	Initial step	Denaturation, annealing, elongation	Cycles
huAnxA8	F	TGG GAC CCT GAT AAG AAA CAT	10 min 95 °C	15 sec 95 °C, 1 min 60 °C	40
	R	TCC TGG AGA CTC TGG CTT CAT			
huCalbindin	F	CCT GCT GCT CTT CCG ATG CCA G			
	R	GGT AGT AAC CTG GCC ATC TCA G			
huCalretinin	F	CCT GCA CCT GGC CGA GCT GAC G			
	R	GCT GGA GCC CAC GTG CTG CCT G			
huGAPDH	F	ACC CAC TCC TCC ACC TTT G			
	R	CTC TTG TGC TCT TGC TGG G			
porcAnxA8	F	CCC AGA CCC CGA CGC GGA GAC C			
	R	GTT CTT GGT CCG AGA AGC CAG G			
porcGAPDH	F	AAG TGG ACA TTG TCG CCA TC			
	R	TCA CAA ACA TGG GGG CAT C			

### AnxA8 overexpression

ARPE-19 cells were transfected at 80–90% confluence using Lipofectamine LTX Plus transfection reagent (Life Technologies). According to the manufacturer’s instructions, 2.5 µg AnxA8-GFP^27^ or GFP plasmid DNA was used to transfect a dish of 8.55 cm² for 6 h at 37°. Afterwards, cells were washed and FR or AnxA8 siRNA treatment was started straight away as described earlier. Phase images were taken after transfection (day 0), on days 3 and 6 of treatment. GFP-transfected cells were counted and quantified as a percentage of the total cell count. Cells were also fixed in 4% PFA after transfection for immunofluorescence analysis to analyse neuronal marker and pHH3 expression, or cells were processed for Western blotting to analyse apoptosis.

### Western blotting

Equal numbers of cells were lysed in 2% sodium dodecyl sulphate (SDS), 25% glycerol, 12.5% 0.5 M Tris-HCl pH 7.4, 0.02% dithiothreitol and 0.02% bromophenol blue at 95 °C for 5 min. Samples were subjected to SDS-polyacrylamide gel electrophoresis using 10% acrylamide gels, and separated proteins were transferred to a nitrocellulose membrane at 30 V for 4 h. Non-specific binding sites were blocked using 7% milk powder in Tris-buffered saline for 1 h, and the membrane was incubated with caspase-3 (1:2000, Cell signalling) and cleaved caspase-3 (1:1000, Cell signalling) antibodies overnight at 4 °C. An HRP-conjugated donkey anti-rabbit secondary antibody (Dako) was applied for 1 h. Proteins were visualised using chemiluminescence (GE Healthcare) and exposed to Kodak Biomax X-ray films. Grey values of protein bands were normalised to those of heat shock chaperone-70 (HSC-70, Santa Cruz, 1:5000), and densitometric analysis was undertaken with ImageJ (NIH freeware).

### Statistical analysis

Data are presented as mean and standard error. Student’s *t*-test was performed for normally distributed data to analyse differences between two groups using Microsoft Excel. Differences with *P* values < 0.05 were considered statistically significant. Sample sizes are indicated in the figure legends.

## Electronic supplementary material

Supplementary Information
