# Supplementary material for: Regulation of retinal pigment epithelial cell phenotype by Annexin A8
Source: Sci Rep. 2017 Jul 5;7:4638. doi: 10.1038/s41598-017-03493-3 (PMC5498634; doi:10.1038/s41598-017-03493-3)

Supplementary Information

Title

Regulation of retinal pigment epithelial cell phenotype by Annexin A8

List of authors

Katharina Lueck[1]*, Amanda-Jayne F. Carr[1,2], Dimitrios Stampoulis[1], Volker Gerke[3], Ursula Rescher[3], John Greenwood[1], and Stephen E. Moss[1].

Figure SI1. Full-length Western blotting images featured in Figure 6D. Please see manuscript for treatments.

Caspase-3 (35 kDa)

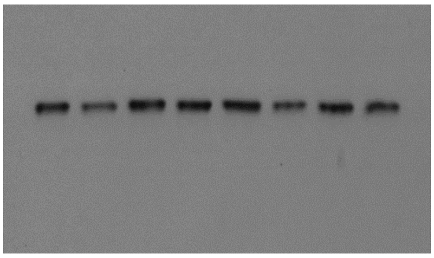

Cleaved caspase-3 (17 kDa)

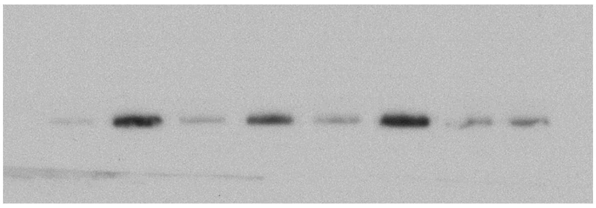

HSC-70 (70 kDa)

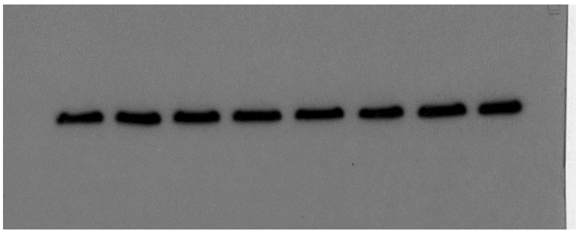

Supplement: Supplementary file 1 — Supplementary Information [file 41598_2017_3493_MOESM1_ESM.pdf]
